# Synergistic effects of sodium butyrate and cisplatin against cervical carcinoma *in vitro* and *in vivo*

**DOI:** 10.3389/fonc.2022.999667

**Published:** 2022-10-21

**Authors:** Huijun Chu, Xiaoyuan Sun, Jia Wang, Ke Lei, Zhengyi Shan, Chenyang Zhao, Ying Ning, Ruining Gong, He Ren, Zhumei Cui

**Affiliations:** ^1^ Department of Obstetrics and Gynecology, The Affiliated Hospital of Qingdao University, Qingdao, China; ^2^ Center of Tumor Immunology and Cytotherapy, Medical Research Center, The Affiliated Hospital of Qingdao University, Qingdao, China; ^3^ Department of Pathology, The Affiliated Hospital of Qingdao University, Qingdao, China; ^4^ Graduate School, Medical College of Qingdao University, Qingdao, China; ^5^ Center for Gastrointestinal (GI) Cancer Diagnosis and Treatment, Tumor Immunology and Cytotherapy, Medical Research Center, The Affiliated Hospital of Qingdao University, Qingdao, China

**Keywords:** cervical cancer, sodium butyrate, cisplatin, epithelial-mesenchymal transition, migration and invasion

## Abstract

**Backgrounds:**

Cisplatin-based chemotherapy has been considered as the pivotal option for treating cervical cancer. However, some patients may present a poor prognosis due to resistance to chemotherapy. As a metabolite of natural products, sodium butyrate (NaB) could inhibit the proliferation of several malignant cells, but little is known about its combination with cisplatin in the treatment of cervical cancer.

**Materials and methods:**

Flow cytometry, CCK-8 assay, and Transwell assay were utilized to analyze the cellular apoptosis, viability, cellular migration and invasion upon treating with NaB and/or cisplatin. The allograft mice model was established, followed by evaluating the tumor volume and necrotic area in mice treated with NaB and/or cisplatin. Western blot was performed for detecting protein expression involved in epithelial-mesenchymal transition (EMT) and the expression of MMPs. Immunohistochemical staining was conducted with the tumor sections. The transcription, expression, and cellular translocation of β-catenin were determined using luciferase reporter gene assay, Real-Time PCR, Western blot, and confocal laser scanning microscope, respectively.

**Results:**

NaB combined with cisplatin inhibited cell viability by promoting apoptosis of cervical cancer cells. *In vivo* experiments indicated that NaB combined with cisplatin could inhibit tumor growth and induce cancer cell necrosis. Single application of NaB activated the Wnt signaling pathway and induced partial EMT. NaB alone up-regulated MMP2, MMP7 and MMP9 expression, and promoted the migration and invasion of cervical cancer cells. The combination of cisplatin and NaB inhibited cellular migration and invasion by abrogating the nuclear transition of β-catenin, reverse EMT and down-regulate MMP2, MMP7 and MMP9. Immunohistochemical staining indicated that NaB combined with cisplatin up-regulated the expression of E-cadherin and reverse the EMT phenotype in the mice model.

**Conclusions:**

NaB serves as a sensitizer for cisplatin, which may be a promising treatment regimen for cervical cancer when combined both. NaB alone should be utilized with caution for treating cervical cancer as it may promote the invasion and migration of cervical cancer cells.

## Introduction

Cervical cancer is the fourth most common female malignancy worldwide, with an estimated 632,320 new cases and appropriately 342,000 deaths, according to the GLOBOCAN 2020 data (1). National Comprehensive Cancer Network (NCCN) clinical practice guidelines in oncology (version 1.2022) recommend definitive chemoradiation for local advanced-stage diseases including the International Federation of Gynecology and Obstetrics (FIGO) stage IB3 and IIA2. Patients with cervical cancer FIGO stages IIB-IVA are also treated with chemoradiation. For patients with stage IVB, adjuvant systemic therapy is considered. Chemotherapy with platinum-containing regimens is administered during radiotherapy, with cisplatin being preferred as a sensitizing agent. Patients with distant metastases and relapse can be treated with systemic therapy, such as the combination of cisplatin/paclitaxel with pembrolizumab with or without bevacizumab for PD-L1-positive tumors. If the patient is cisplatin intolerant, carboplatin can be used as an alternative drug.

Cisplatin plays an important role in the treatment of cervical cancer, especially in concurrent chemoradiotherapy and systemic chemotherapy. Cisplatin ((SP-4-2)-diamminedichloridoplatinum(II)), one of the best metal-based chemotherapeutic drugs, exerts anticancer activity by interacting with purine bases on genomic DNA or mitochondrial DNA (2). However, the prognosis is still poor with a five-year survival of less than 3% among the patients at stage III and IV (3, 4). Moreover, there are still some reports of adverse events such as gastrointestinal toxicity, hepatotoxicity, nephrotoxicity, ototoxicity, renal toxicities, as well as bone marrow depression (5, 6). Besides its side effects, the drug resistance of cisplatin limits its effectiveness and application. Therefore, it is urgent to develop new strategies to achieve high survival rates with low toxicities.

In recent years, natural dietary compounds have gained increasing attention as adjuvant therapy due to relatively low toxicity and synergistic effects with current chemotherapeutic agents (7). Black raspberries (BRBs), which function as inhibitors of cancer or premalignancy in high-risk human cohorts, were reported to inhibit proliferation and promote apoptosis of cervical cancer cells (8). As an active metabolite of BRBs, butyrate could inhibit the growth of many malignant cancer cells (9). Sodium butyrate (NaB), as an inhibitor of histone deacetylases (HDACs), has been considered as a promising cisplatin sensitizer of cancer cells (10). It could inhibit proliferation and promote apoptosis *in vivo* and *in vitro* (11). NaB also relieves cisplatin-induced adverse events such as hearing loss, renal inflammation, and gut microbiota disorder (12, 13).

However, there are some studies reporting that NaB induces epithelial-mesenchymal transition (EMT) in hepatocellular carcinoma (HCC) (14). Additionally, NaB triggers migration and invasion of oral squamous cell carcinoma (15). These paradox results lead us to investigate the efficiency of NaB or its combination with cisplatin in cervical cancer cells. This study is also aimed to explore the potential mechanism of NaB in combination with cisplatin in this process, which may be used to enhance the therapeutic effects of cisplatin and mitigate side effects.

## Materials and methods

### Cell culture

Cervical cancer HeLa cell line was purchased from the Chinese Academy of Sciences Cell Bank (Shanghai, China). Cervical cancer Siha cells were obtained from Procell Life (Wuhan, China). Cells were grown in DMEM/MEM supplemented with 10% fetal bovine serum (FBS) and 1% penicillin-streptomycin. Hela and Siha cells were cultured in a humidified incubator containing 5% CO_2_ at 37°C. The cells were allowed to adhere overnight. This study protocol was obtained the approval from the Ethics Committee of the Affiliated Hospital of Qingdao University (No. QYFY WZLL 27060).

### Cytotoxicity assay

Hela and Siha cells were seeded in 96-well plates. The cells were collected for the subsequent analysis upon a confluence of 50%-60%. To evaluate the half-maximal inhibitory concentration 50 (IC50) of NaB, cells were incubated with a concentration of 1 mM, 2 mM, 4 mM, 8 mM and 16 mM for 24 h, 48 h, and 72 h. To detect the cytotoxicity of NaB in combination with cisplatin, Hela and Siha cells were incubated with NaB (1 mM, 2 mM, and 4 mM) alone or in combination with cisplatin. Cellular viability was determined with 10% CCK-8 reagent according to the manufacturer’s instructions (Meilunbio, Dalian, China). The absorbance was detected using a microplate reader at 450 nm. The cell viability of the drug group was shown as percentage of the control group set to 100%.

### Apoptosis assay

Fluorescein isothiocyanate (FITC)-Annexin V kit was used to detect the apoptotic rate of Hela and Siha cells. Hela cells were incubated simultaneously with 4 mM NaB and 1 μg/mL cisplatin for 24 h, and Siha cells for 48 h. The cells (1 × 10^4^) were collected and washed in pre-cold phosphate buffer saline twice and resuspended in 100 μL Binding Buffer (1 ×). Then cervical cancer cells were treated with 2.5 μL propidium iodide and 2.5 μL FITC-Annexin V and incubated for 15 min. Upon treatment, 400 μL binding Buffer was added. The apoptotic cells were analyzed using a Beckman Coulter Cytomics FC 500 flow cytometer (Beckman Coulter, Brea, CA, USA).

### Transwell assay

Cervical cancer cells were pretreated simultaneously with 4 mM NaB and/or 1 μg/mL cisplatin, respectively. The cells treated with double distilled water served as the negative control. The cells were incubated for 12 h. The cells were resuspended in serum-free medium and planted in the upper chambers (5 × 10^4^ HeLa and 10 × 10^4^ Siha per well). The lower chamber was added with DMEM containing 10% FBS and 1% antibiotics. The cells were cultivated in a humidified incubator containing 5% CO_2_ at 37°C for 24 h. To evaluate the cellular invasion, the chamber was coated with Matrigel according to the manufacturer’s instructions. Upon the removal of non-migrating or non-invading, the remaining cells were fixed and were stained with 0.05% crystal violet solution. Finally, the cells in five randomly selected fields were observed under the Olympus microscope (Olympus, Tokyo, Japan).

### Allograft model and drug treatment

The experiments involving animals were approved by the Ethics Committee of the Affiliated Hospital of Qingdao University. Four-week-old Balb/c nude mice (weight: 18-20 g) were purchased from Vital River (Beijing, China). The mice were maintained under pathogen-free conditions with a photoperiod of 12 of light. Hela cells were subcutaneously into the axilla of female nude mice. The tumor volume was calculated in (length × width^2^)/2 per week. Mice with tumor volume among 100-200 mm^3^ were randomly grouped into the control (n = 6), NaB (n = 6), cisplatin (n = 6), and NaB in combination with cisplatin (n = 6) groups. Mice were injected with NaB (200 mg/kg/day) for 5 consecutive days per week in NaB group, or cisplatin (5 mg/kg/week) for 3 weeks in the cisplatin group *via* intraperitoneal injection. Mice in the combination group mice were injected with NaB (200 mg/kg/day) and cisplatin (5 mg/kg/week) *via* intraperitoneal injection. Mice in the control group were injected with normal saline *via* intraperitoneal injection. Subcutaneous tumors were harvested for immunohistochemical analysis.

### Immunohistochemistry analysis

The specimens were fixed in formalin, followed by embedding in paraffin. The sections (4 μm) were deparaffinized in xylene, rehydrated in ethanol with decreasing graded concentrations, and stained with hematoxylin and eosin. The slides were pressure cooked with citrate buffer (pH = 6.0) for 10 min. Endogenous peroxide activity was quenched by incubating the slides with 3% H_2_O_2_ for 20 min. The slides were blocked with goat serum at 37°C for 30 min. The primary antibodies were incubated with antibodies against E-cadherin, N-cadherin, and vimentin for 2 h. The secondary antibody was then incubated with the slides at 37°C for 30 min, followed by the visualization with DAB. The slides were mounted with permanent mounting media after hematoxylin counterstaining. Images were captured under a microscope.

### EMT assay

Hela and Siha cells (1 × 10^5^ cells/each well) were inoculated in 6-well plates. The cells were simultaneously treated with 4 mM NaB and 1 μg/mL cisplatin for 24 h. After removing the medium, the cells were washed gently with phosphate-buffered saline for 3 times. The cells were fixed using methanol and stained with crystal violet solution for 20 min. The excess crystal violet solution was washed off using phosphate-buffered saline. The cell morphology was observed under a microscope.

### Fractionation of cytoplasmic and nuclear proteins

Cytoplasmic and nuclear proteins were extracted using the nuclear protein extract reagent kit from Solarbio (Beijing, China). Phenylmethylsulfonyl fluoride (PMSF) was mixed with the cytoplasmic protein extract reagent or nuclear protein reagent to obtain PMSF solution at a concentration of 1 mM. The cells were washed in PBS and digested with EDTA. Thereafter, the cells were collected by centrifugation (500 g, 3 min). Then 20 μL pellets were dissolved in 200 μL of cytoplasmic protein extract reagent, followed by vortex agitation for 15 s. The mixture was maintained on ice for 10 min and vibrated for 10 s, followed by centrifugation (12,000 g, 10 min). The supernatant collected was the cytoplasmic proteins. The pellets were added with 100 μL nuclear protein extract reagent and the mixture was adequately mixed. The mixture was maintained on the ice for 10 min, and then vibrated for 10 s. The supernatant was collected by centrifugation (12,000 g, 10 min) and was used as nuclear proteins.

### Quantitative RT-PCR

Hela and Siha cells were plated in 6-well plates with 2 × 10^5^ cells each well. The cells were simultaneously treated with 4 mM NaB and 1 μg/mL cisplatin (Hela cells for 24 h and Siha cells for 48 h). The cells were collected and washed in pre-cold phosphate-buffered saline after the treatment. Total RN was extracted using TRIZOL reagent (Invitrogen, Carlsbad, CA, USA). Evo M-MLV One-Step RT-PCR Kit (AG, Changsha, China) was used for reverse transcription. qRT-PCR was carried out using SYBR Green Premix Pro Taq HS Premix on LightCycler 96 (Roche, Penzberg, Germany). *GAPDH* was used as the housekeeping gene. The primers were listed in **Table 1**.

**Table 1:** Oligonucleotide primers used for qRT-PCR.

Gene	Forward (5’ to 3’)	Reverse (5’ to 3’)
β-catenin	AAGGTGTGGCGACATATGCA	CAAGTCCAAGATCAGCAGTCTCA
GAPDH	CATGTTCGTCATGGGTGTGAA	GGCATGGACTGTGGTCATGAG

### Western blot

Hela and Siha cells were collected after simultaneous incubation with 4 mM NaB and 1 μg/mL cisplatin. Hela cells were treated for 24 h, Siha cells for 48 h. Whole proteins were extracted using RIPA lysis buffer for 30 min on ice and then scraped immediately. Appropriately 30-50 μg of total proteins were separated using 10% SDS-PAGE. The separated proteins were transferred onto the PVDF membrane, and 5% non-fat milk was used to block the membrane for 1 h at room temperature. Primary antibodies against Bcl-2, Bax, E-cadherin, N-cadherin, vimentin, MMP2, MMP7, MMP9, β-catenin, Lamin B, β-actin, and GAPDH were incubated with the membrane overnight at 4°C. The secondary antibody marked by horseradish peroxidase was used to detect primary antibody-protein complexes at room temperature for 1 h. The proteins were detected by enhanced chemiluminescence solution. The signal intensity was quantified using Image J software.

### Luciferase reporter gene assay

Hela and Siha cells were planted in 24-well plates. The cells were simultaneously treated with 4 mM NaB and 1 μg/mL cisplatin for 24 h. After removing the medium, the cells were transfected with *CTNNB1* promoter linked to GV238 plasmid carrying the luciferase reporter gene (MCS-firefly_Luciferase) or CON245 plasmid harboring the TK promoter-Renilla luciferase. Lipofectamine 3000 (Invigrogen) was used as the transfection reagent. Dual-Luciferase Reporter 1000 Assay system (Promega, Madison, WI, USA) was used to detect luciferase activities 24 h after transfection according to the instructions.

### Confocal microscopy

Hela and Siha cells were grown on slides and simultaneously treated with 4 mM NaB and 1 μg/mL cisplatin. Hela cells were treated for 24 h, and Siha cells were treated for 48 h. Then the cells were fixed in 4% paraformaldehyde for 30 min and immersed in goat serum for 30 min at room temperature. The cells were then incubated with anti-β-catenin antibody (1:80) overnight at 4°C. After washing in the immune-staining washing solution, the cells were incubated with secondary anti-rabbit antibody (1:1,000) for 1 h at room temperature. Next, the cells were washed with the immune-staining washing solution and incubated with DAPI (10 mg/mL) in phosphate-buffered saline for 10 min. β-catenin localization was analyzed using a confocal laser scanning microscope (Olympus).

### Statistical analysis

Statistical significance of cell viability, apoptosis rate, and gene expression was evaluated by two-tailed unpaired Student’s *t* test and one-way ANOVA followed by Bonferroni test. GraphPad Prism Software Version 9.0 (GraphPad, La Jolla, CA, USA) was used for statistical analysis. All data were expressed as the mean + standard deviation from 3 independent experiments.

## Results

### NaB combined with cisplatin inhibited the cell viability of Hela and Siha cells through promoting their apoptosis

**Figure 1:**
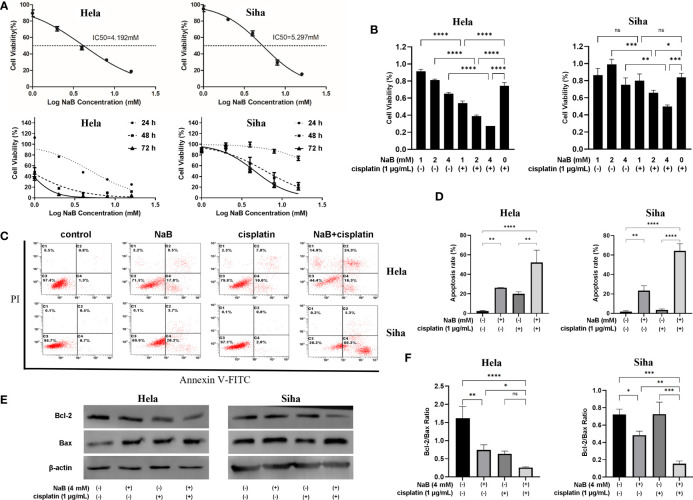
NaB combined with cisplatin inhibited the cell viability of Hela and Siha cells through promoting their apoptosis. **(A)** Cell viability was detected 24 h, 48 h and 72 h after Hela and Siha cells were treated with NaB (1, 2, 4, 8 and 16 mM). **(B)** The viability of Hela and Siha cells was examined after the treatment with NaB (1, 2, and 4 mM) alone or in combination with cisplatin (1 μg/mL). **(C)** Annexin V-FITC- and PI- stained cells were detected by flow cytometer, and **(D)** apoptosis rates of Hela and Siha cells are quantitatively shown. **(E, F)** Protein bands of Bcl-2, Bax and β-actin (internal control) were visualized by Western blot and the expression was quantitatively presented. Hela cells were examined 24 h and Siha cells were examined 48 h after the treatment with NaB (4 mM) alone or in combination with cisplatin (1 μg/mL) **(B–F)**. Data represent the mean + standard deviation from 3 independent experiments. Significance was examined by one-way ANNOVA (^ns^P>0.05, *P<0.05, **P<0.01, ***P<0.001, ****P<0.0001). NaB, sodium butyrate; FITC, fluorescein isothiocyanate; PI, propidium iodide.

CCK-8 assay indicated that the IC_50_ of NaB was 4.192 mM for Hela cells and 5.297 mM for Siha cells. NaB could inhibit the viability of Hela cells and Siha cells in a time- and dose-dependent manner (**Figure 1A**). The combination of NaB (4 mM) and cisplatin (1 µg/ml) could significantly inhibit the cellular viability compared with that of single application of cisplatin (P<0.05, **Figure 1B**). We then examined whether the inhibitory effects of NaB and cisplatin on cellular viability were associated with apoptosis. Our data showed that the combination of NaB and cisplatin could significantly enhance the apoptosis of Hela cells and Siha cells, compared with the control group (P<0.05) or single cisplatin group (P<0.01) (**Figures 1C, D**). Bcl-2 protein in Hela and Siha cells was down-regulated after treated with NaB combined with cisplatin compared with that of the control group or cisplatin group. Bcl-2/Bax ratio was significantly reduced in Siha cells treated with NaB in combination with cisplatin compared to single application of NaB (P<0.05) or cisplatin (P<0.05). Bcl-2/Bax ratio in Hela cells was significantly decreased by the combination of NaB and cisplatin (**Figures 1E, F**). However, it showed no significant differences compared with that in the cisplatin group.

### NaB combined with cisplatin inhibited migration, invasion and EMT of cervical cancer cells

**Figure 2:**
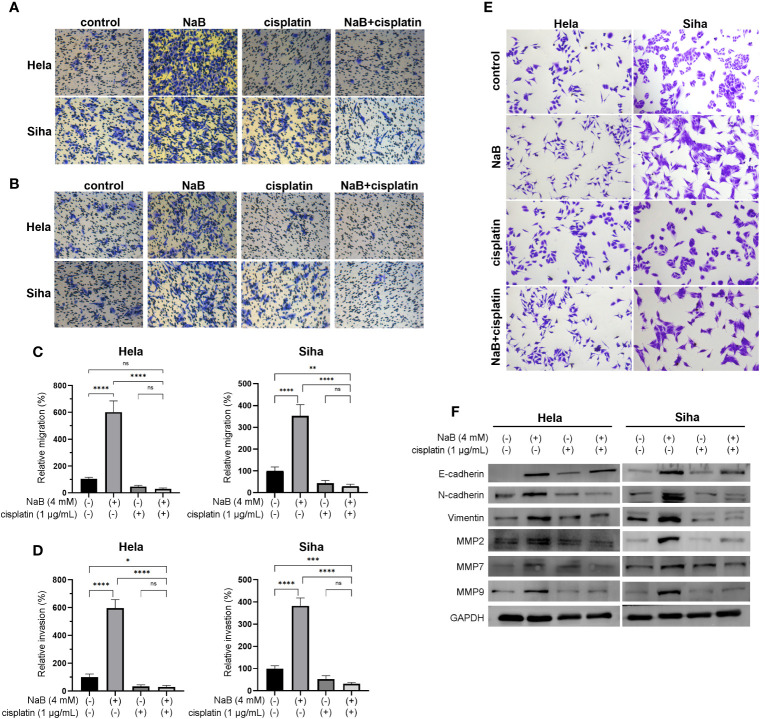
NaB combined with cisplatin inhibited the cell viability of Hela and Siha cells through promoting their apoptosis. **(A)** Migration and **(B)** invasion were detected by Transwell assays. **(C)** Migration and **(D)** invasion were quantitatively presented in the histogram. **(E)** Spindle-shaped morphological changes of Hela and Siha cells were observed after crystal violet staining. **(F)** Protein bands of E-cadherin, N-cadherin, vimentin, MMP2, MMP7, MMP9, and GAPDH (internal control) were visualized by Western blot. Hela and Siha cells were treated with NaB (4 mM) alone or in combination with cisplatin (1 μg/mL). Data represent the mean + standard deviation from 3 independent experiments. Significance was examined by one-way ANNOVA (^ns^P > 0.05, *P < 0.05, **P <0.01, ***P < 0.001, ****P < 0.0001). NaB, sodium butyrate. Magnification: 200 ×.

In this part, we analyzed the effects single application of NaB or its combination with cisplatin on invasion, migration and EMT of cervical cancer cells. Transwell assay indicated that NaB induced significant migration and invasion of HeLa and Siha cells compared with that of control. The migration and invasion were significantly inhibited in the cells treated with the combination of NaB and cisplatin compared that of control and NaB group (**Figures 2A–D**). Cervical cancer cells treated with NaB showed spindle-shaped morphological changes, which indicated the presence of EMT. In contrast, no change in cell morphology was observed in the NaB combined with cisplatin group (**Figure 2E**). This morphological alteration suggested the combination of NaB and cisplatin did not induce EMT in cervical cancer cells. Western blot was conducted to investigate the roles of NaB in the EMT process in these cells. Compared with the control, the expression of EMT-related protein including E-cadherin, N-cadherin, and vimentin in NaB group was significantly up-regulated *in vitro*. These results indicated the possibility of partial EMT after NaB treatment. However, the combination of cisplatin and NaB induced significant up-regulation of E-cadherin and down-regulation of N-cadherin and vimentin (**Figure 2F**). This was consistent with the morphological findings.

MMPs played a pivotal role in the cellular migration and invasion. In addition, over-expression of MMPs and gelatinase was closely associated with EMT. On this basis, we determined whether NaB could regulate the expression of MMPs in the cervical cancer cells. Similarly, the expression of MMP2, MMP7, and MMP9 in the NaB group showed a significant increase compared with the control group. However, NaB + cisplatin group showed a significant decrease compared with that in the NaB group. The expression of MMP2, MMP7, and MMP9 in the NaB + cisplatin was not statistically different from that of the cisplatin group (**Figure 2F**). This indicated that the up-regulation of MMP2, MMP7, and MMP9 induced by NaB could be inhibited when combing with cisplatin.

### *In vivo* effects of NaB alone or in combination with cisplatin on cervical cancer

**Figure 3:**
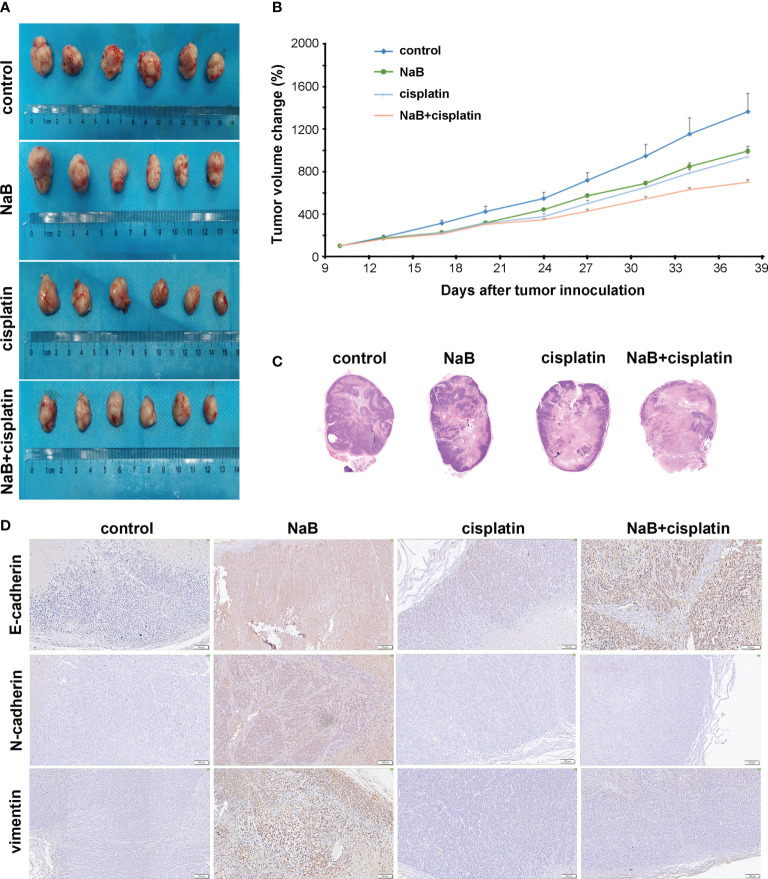
*In vivo* effects of NaB alone or in combination with cisplatin on cervical cancer. **(A, B)** The tumor volume of the mouse xenografts. **(C)** Representative H&E staining of subcutaneous xenografts (Magnification: 200 ×). **(D)** Immunohistochemical staining of E-cadherin, N-cadherin, and vimentin in tumor tissues collected from mouse models of cervical cancer 4 weeks after the treatment. Bars: 100 μm. The mice were treated with NaB alone (200 mg/kg/day) or in combination with cisplatin (5 mg/kg/week). NaB, sodium butyrate.

A mouse model of cervical cancer was constructed and treated with NaB and/or cisplatin, respectively. NaB combined with cisplatin was obviously more effective than either control, NaB, or cisplatin in inhibiting the tumor volume (**Figures 3A, B**). H&E staining showed that the size of necrotic fraction tended to be larger in the NaB combined with cisplatin group as compared to NaB group or cisplatin group (**Figure 3C**). Then immunohistochemical staining was conducted to determine E-cadherin, N-cadherin, and vimentin levels. The results showed that NaB promoted the expression of E-cadherin, N-cadherin, and vimentin in tumor tissues. However, compared with the control group, the combination of NaB and cisplatin promoted the expression of E-cadherin, rather than N-cadherin, and vimentin (**Figure 3D**). These were consistent with the *in vitro* experiments. This implied that NaB combined with cisplatin inhibited tumor growth, and reversed EMT process through up-regulating E-cadherin.

### Effects of NaB in combination with cisplatin on Wnt/β-catenin signaling

**Figure 4:**
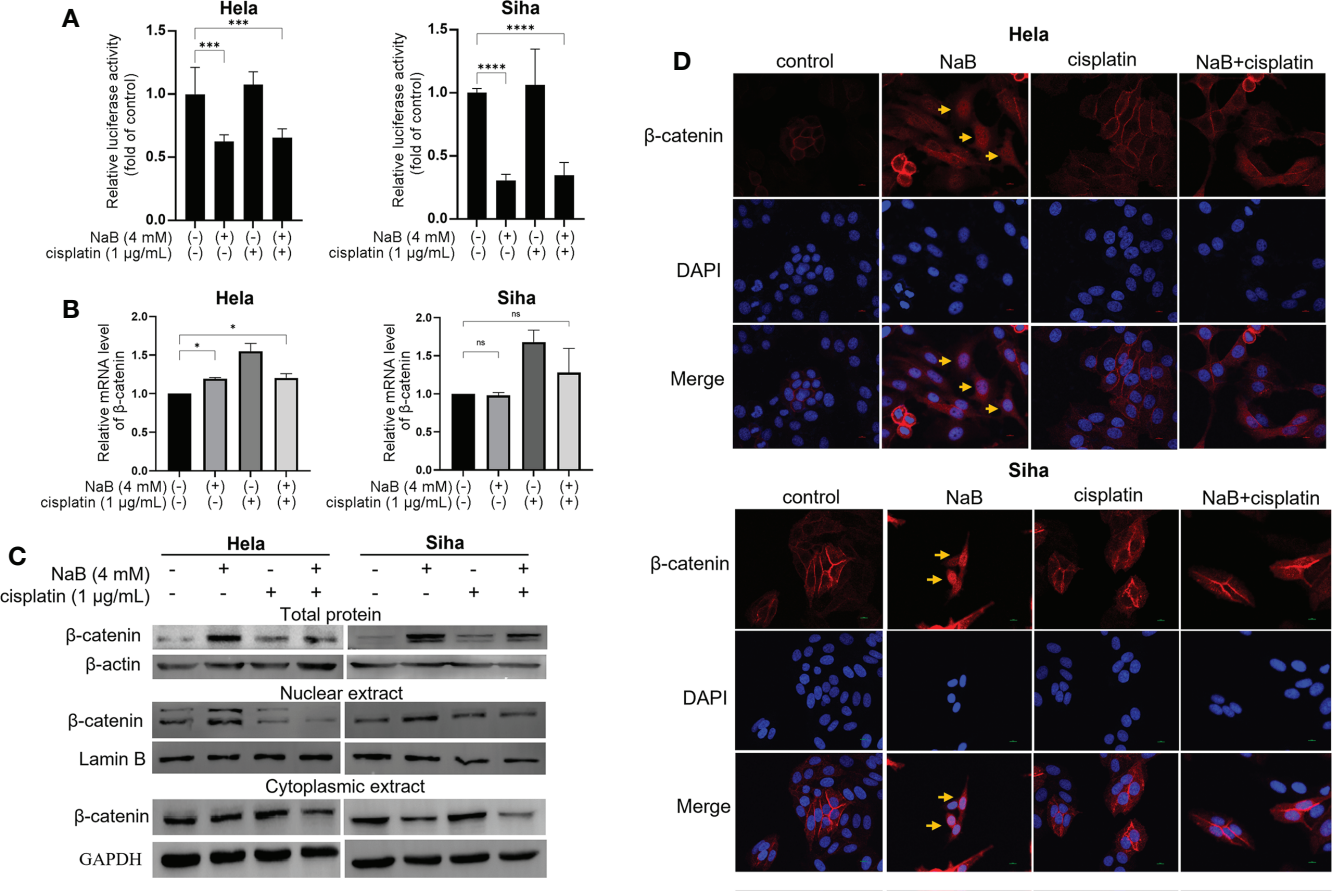
Effects of NaB in combination with cisplatin on Wnt/β-catenin signaling. **(A)** The luciferase activity in Hela and Siha cells was measured by luciferase reporter gene assay. **(B)** qRT-PCR was applied to detect the relative expression of β-catenin. **(C)** Protein bands of β-catenin from total protein, nuclear extract and cytoplasmic extract in Hela and Siha cells were visualized. **(D)** Immunofluorescence analysis of β-catenin (red) in Hela and Siha cells. Arrowhead (yellow) indicates the accumulation of β-catenin in the nuclear region. Hela and Siha cells were treated with NaB (4 mM) alone or in combination with cisplatin (1 μg/mL). Data represent the mean + standard deviation from 3 independent experiments. Significance was examined by one-way ANOVA (^ns^P > 0.05, *P < 0.05, ***P < 0.001, ****P < 0.0001). NaB, sodium butyrate. Magnification: 200 ×.

Wnt/β-catenin is a classical signaling pathway modulating cancer cell migration, invasion and EMT process. The activation of Wnt/β-catenin pathway is marked by the accumulation and translocation of β-catenin in the nucleus. For the dual luciferase assay, NaB inhibited the promoter activity of β-catenin in cervical cancer cells whether it was used alone or in combination with cisplatin (**Figure 4A**). Results from qRT-PCR further verified that NaB alone or in combination with cisplatin conferred no significant effects on the transcription of β-catenin mRNA (**Figure 4B**). We speculated that NaB or its combination with cisplatin may involve in regulating the downstream targets through modulating the translation of β-catenin. First, Western blot was utilized to determine the β-catenin in total protein. NaB significantly up-regulated the expression of β-catenin in total protein, while its combination with cisplatin could down-regulate the expression of β-catenin in total protein. Then the nuclear protein and cytoplasmic protein were separated. Our results showed that NaB significantly up-regulated the expression of nuclear β-catenin in Hela and Siha cells. In contrast, the protein expression of nuclear β-catenin was reduced after the simultaneous treatment by NaB and cisplatin (**Figure 4C**). Then the localization of β-catenin protein was observed using immunofluorescence confocal microscopy. The results showed that β-catenin translocated from the cell membrane to the nucleus after treating with NaB, resulting in accumulation of β-catenin in the nucleus in Hela and Siha cells. In contrast, the nuclear translocation of β-catenin was significantly inhibited in the cells treated with NaB combined with cisplatin, compared with single application of NaB (**Figure 4D**). This indicated that NaB could trigger the nuclear translocation of β-catenin serving as a hallmark for activating the Wnt/β-catenin signaling. However, the combination of NaB and cisplatin could significantly inhibit the translocation of β-catenin from membrane to the nucleus.

### Wnt pathway inhibitor SM04690 relieved the effects of NaB on migration, invasion, and EMT process

**Figure 5:**
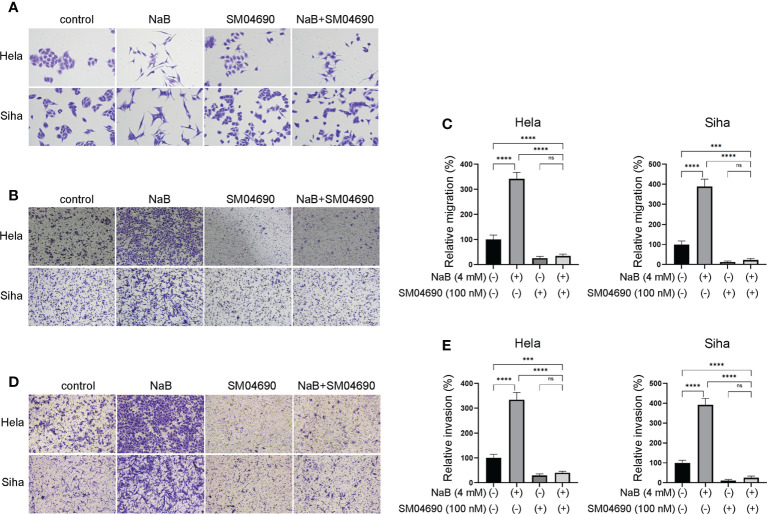
Wnt pathway inhibitor SM04690 relieved the effects of NaB on migration, invasion, and EMT process. **(A)** Spindle-shaped morphological changes of Hela and Siha cells were observed after crystal violet staining. **(B, C)** Migration and **(D, E)** invasion were detected by Transwell assays. Hela and Siha cells were treated with NaB (4 mM) alone or in combination with SM04690 (100 μM). Significance was examined by one-way ANNOVA (^ns^P > 0.05, ***P < 0.001, ****P < 0.0001). NaB, sodium butyrate. Magnification: 200 ×.

We further investigated whether NaB could modulate migration, invasion, and EMT process through Wnt signaling pathway. Hela and Siha cells were treated by Wnt signaling pathway inhibitor SM04690. NaB induced spindle-shaped changes in cervical cancer cells, while the addition of SM04690 reversed the effect of NaB (**Figure 5A**). The results suggested that the blocking of Wnt signaling pathway by SM04690 prohibited the NaB-induced cell morphology alteration. Transwell assay showed that NaB alone significantly induced the migration and invasion of cervical cancer cells (**Figures 5B–E**). However, the addition of SM04690 decreased the migration and invasion of these cells by NaB. These suggested that NaB modulated migration, invasion and EMT process in Hela cells and Siha cells were mainly based on targeting the Wnt signaling pathway.

## Discussion

The cisplatin-based chemotherapy has been commonly utilized for treating patients with cervical cancer (16, 17). However, many cases present poor prognosis due to recurrence, adverse events, and chemotherapeutic drug resistance (16, 17). Therefore, some efforts have been taken to find new natural anticancer agents with low toxicity and high efficacy. Recently, NaB serving as an intestinal flora derivative has been utilized as it shows general anti-cancer effects (18, 19). However, its efficiency is limited for treating the epithelium-derived malignancies, and the mechanism is still not well defined. Moreover, the treatment efficiency may be different when combing with different anti-cancer agents with varying targets. NaB combined with cisplatin has been reported to be effective for killing cancer cells under *in vitro* conditions (20). In the present study, the combination of NaB with cisplatin enhanced the sensitivity of Hela and Siha cells to cisplatin. Besides, NaB in combination with cisplatin inhibited cell migration, invasion and EMT under *in vivo* and *in vitro* conditions. This suggested its potential application as an effective therapeutic strategy.

The majority of studies on NaB indicated that it could induce sensitization of cancer cells to cisplatin, including ovarian cancer cells (21), gastric cancer cells (22), and bladder cancer cells (20). Specifically, NaB has been reported to show anti-cancer effects on human oral mucoepidermoid carcinoma through modulating the caspase-dependent apoptosis (23). In addition, it could promote apoptosis of breast cancer cells through producing reactive oxygen species and triggering mitochondrial impairment (18). Few studies had focused on the roles of the combination of NaB and cisplatin for treating cervical cancer. In a previous study, Li et al. reported that NaB combined with cisplatin increased the apoptosis of gastric cancer cells *via* the mitochondrial apoptosis pathway (22). In addition, the NaB could enhance the cytotoxic effects of cisplatin in Hela cells (24). Consistent with these findings, the cytotoxic effect of cisplatin on cervical cancer cells was enhanced by NaB as verified by apoptosis assay. The cytotoxic effect of NaB was related to the induction of apoptosis, which was proved by the decreased ratio of Bcl-2 to Bax. Nevertheless, NaB was also considered as a double-edged sword in cancer pathogenesis. NaB was shown to promote EMT in HCC *via* the AMPK-FOXO1-ULK1 signaling axis-mediated autophagy, which then resulted in poor prognosis among these patients (14). Meanwhile, NaB could promote the metastasis of hepatoma cells, and trigger the invasion and migration of cancer cells (25). In this study, NaB promoted the migration and invasion of cervical cancer cells. Moreover, NaB triggered partial EMT that was considered as an important hallmark for drug resistance. Under *in vivo* conditions, NaB promoted partial EMT process, featured by up-regulation of E-cadherin, N-cadherin, and vimentin in tumor tissues.

EMT has been considered to be closely related to the resistance to cisplatin-based chemotherapy (26). Cancer cells undergoing EMT usually show down-regulation of E-cadherin, while the morphological changes are usually accompanied with up-regulation of vimentin and N-cadherin (27, 28). Besides, EMT is closely related to over-expression of MMP2 and MMP9 that are also correlated with cancer cell invasion and metastasis (29–32). Our study revealed that NaB significantly up-regulated the expression of MMP2, MMP7, and MMP9, as well as N-cadherin and vimentin. Morphological changes and the expression of N-cadherin and vimentin were typical signs of mesenchymal transition. However, the expression of MMP2, MMP7, MMP9, N-cadherin and vimentin was significantly down-regulated in the cells treated with the combination of NaB and cisplatin, while the expression of E-cadherin was still up-regulated. *In vivo* experiments demonstrated that NaB induced up-regulation of E-cadherin, N-cadherin and vimentin, indicating the occurrence of partial EMT. However, the combination of NaB with cisplatin up-regulated the expression of E-cadherin but down-regulated the expression of N-cadherin and vimentin, indicating reversal of EMT. Our data showed that NaB induced the migration and invasion of cervical cancer cells, together with incomplete EMT. This was eliminated when combing with the cisplatin.

β-catenin plays important roles in the interaction with the cadherins at the cell junction (33). Free β-catenin could enter into the nucleus, which then regulated the gene expression (34). Our data indicated that NaB could induce the nuclear translocation of β-catenin, which was a hallmark of the activation of Wnt pathway. β-catenin is involved in the regulation of EMT. Upon the combination with cisplatin, β-catenin was mainly expressed in the cellular membrane, which could enhance the cellular adhesion. In this study, SM04690 served as an inhibitor of Wnt signaling pathway, which was utilized to analyze the functional mechanism of NaB. Our data showed that SM04690 blocked the morphological changes triggered by NaB, together with its effects on the migration and invasion of cancer cells. This indicated that Wnt signaling pathway was mediated by NaB and played a crucial role in EMT process.

To date, much attention has been paid to the anti-cancer capacity of natural extracts on several malignancies when utilizing with the conventional chemotherapeutic agents. For example, the combination of naturally sourced compounds with the conventional chemotherapeutic agents reduces side effects, improves sensitization and decreases resistance (35). Therefore, enormous efforts have been made to find natural anticancer products with high response and low drug resistance, which are clinically meaningful. Previous studies showed that BRBs extract functions as an inhibitor of cancer by inhibiting proliferation and regulating the apoptosis of several malignancies such as cervical cancer cells (8, 36). For instance, BRBs extract had been reported to show chemo-preventive effects in patients with colorectal cancer (9, 37), or ApcMin/+ mice with colonic adenoma (38, 39). In this study, NaB combined with cisplatin induced sensitization of cancer cells to cisplatin, and reversed the EMT phenotype through up-regulating the E-cadherin. These indicate the promising utilization of the herbal extract when combing with conventional chemotherapeutic agents.

Indeed, there are some limitations. First of all, we did not construct an animal model of *in situ* and metastasis due to technical limitations. Second, we did not establish an animal model of precancer or HPV infection to evaluate the preventive effects of NaB in the pathogenesis of cervical cancer. Our future direction will focus on the abovementioned animal models to further examine the *in vivo* effects of NaB in enhancing cisplatin-based chemotherapy. This will provide deeper insights into the potential application of NaB in treating cervical cancer.

## Conclusions

As a metabolite of natural product, NaB enhanced the cytotoxicity of cisplatin to the cervical cancer cells. The combination of NaB and cisplatin would inhibit the nuclear transition of β-catenin and reverse the EMT. Serving as a sensitizer for cisplatin, NaB may serve as a promising option for treating cervical cancer. However, its application alone should be cautious as it may induce migration, invasion and partial EMT.

## Data availability statement

The original contributions presented in the study are included in the article/supplementary material. Further inquiries can be directed to the corresponding authors.

## Ethics statement

The animal study was reviewed and approved by Ethics Committee of the Affiliated Hospital of Qingdao University (No. QYFY WZLL 27060).

## Author contributions

HR and ZC contributed to conception and design of the study. HC, XS, JW and KL organized the database. ZS, CZ, YN and RG performed the statistical analysis. HC wrote the first draft of the manuscript. HR and ZC wrote sections of the manuscript. All authors contributed to manuscript revision, read, and approved the submitted version.

## Funding

This work was supported by the Natural Science Foundation of Shandong Province (Grant No. ZR2019MH121 and ZR2020ZD11); the National Science Fund for Distinguished Young Scholars Fund (Grant No. 82125026), the Taishan Scholars Program of Shandong Province (Grant No. Ts20190987); and the Tianjin Medical University Cancer Institute and Hospital (No. 18JCJQJC47800).

## Conflict of interest

The authors declare that the research was conducted in the absence of any commercial or financial relationships that could be construed as a potential conflict of interest.

## Publisher’s note

All claims expressed in this article are solely those of the authors and do not necessarily represent those of their affiliated organizations, or those of the publisher, the editors and the reviewers. Any product that may be evaluated in this article, or claim that may be made by its manufacturer, is not guaranteed or endorsed by the publisher.
